# Advances in engineering microbial biosynthesis of aromatic compounds and related compounds

**DOI:** 10.1186/s40643-021-00434-x

**Published:** 2021-09-27

**Authors:** Roman M. Dickey, Amanda M. Forti, Aditya M. Kunjapur

**Affiliations:** Department of Chemical & Biomolecular Engineering, University of Delaware, Newark, USA

**Keywords:** Metabolic engineering, Biocatalysis, Synthetic biology, Aromatic, Modular pathway design, Co-culture, Aldehydes, Lignin utilization, Plastic utilization, Non-standard amino acids

## Abstract

Aromatic compounds have broad applications and have been the target of biosynthetic processes for several decades. New biomolecular engineering strategies have been applied to improve production of aromatic compounds in recent years, some of which are expected to set the stage for the next wave of innovations. Here, we will briefly complement existing reviews on microbial production of aromatic compounds by focusing on a few recent trends where considerable work has been performed in the last 5 years. The trends we highlight are pathway modularization and compartmentalization, microbial co-culturing, non-traditional host engineering, aromatic polymer feedstock utilization, engineered ring cleavage, aldehyde stabilization, and biosynthesis of non-standard amino acids. Throughout this review article, we will also touch on unmet opportunities that future research could address.

## Introduction

Aromatic compounds function in roles as diverse as flavors, dyes, neurotransmitters, therapeutics, and monomers for polymeric materials. In addition to serving as end products, aromatic compounds find many uses as building blocks in the chemical industry. Over 40% of bulk chemicals are estimated to contain an aromatic component (Haveren et al. 2008). Historically, most aromatic chemicals are derived from fossil fuels, which are converted to benzene, toluene, xylene, or other simple aromatic compounds by several methods. These methods include the reforming of naphtha, which is a mostly non-aromatic C_6_–C_12_ hydrocarbon mixture, the pyrolysis of hydrocarbons during ethylene manufacturing, the catalysis of light hydrocarbons into aromatic compounds, and the pyrolysis of coal (Sweeney and Bryan 2000). However, as society is increasingly prioritizing sustainable routes to chemical manufacturing and is shifting away from its dependence on fossil fuels, there are more opportunities for the production of aromatic compounds from renewable resources. In addition to the interest in sustainable sources of aromatic bulk chemicals, an increasing awareness of the bioactive properties of aromatic natural products is a strong driver for the development of engineered fermentative aromatic synthesis.

Here, we strive to capture the latest innovations in the arena of microbial aromatic compound biosynthesis. Many excellent reviews have already been published on this topic, including one that similarly focuses on emerging trends rather than specific pathways and products (Machas et al. 2019). Another outstanding review provides a detailed description of pathways and products resulting from aromatic metabolism (Cao et al. 2020). As such, here we take more of the former approach of discussing trends, with a focus on strategies that have been reported during the last 5 years or that appear more relevant for future directions. We focus on heterologous reactions and do not describe strategies to increase flux from central metabolism that have been covered in detail by others (Berry 1996). Additionally, we do not discuss all relevant strategies from metabolic engineering; for example, we chose to exclude metabolite biosensors. Extensive work has been performed during the last decade for the identification and engineering of genetically encoded metabolite biosensors based on allosteric transcription factors. We direct readers who may be interested in that topic towards other excellent reviews (Machas et al. 2019; Alvarez-Gonzalez and Dixon 2019). With the exception of certain key steps in flavonoid and stilbenoid biosynthesis, we also do not discuss aromatic polyketide biosynthesis as the assembly of these compounds can deviate significantly from the biosynthetic pathways of most aromatic compounds (Shen 2000).

### Where do aromatic metabolites come from?

**Fig. 1:**
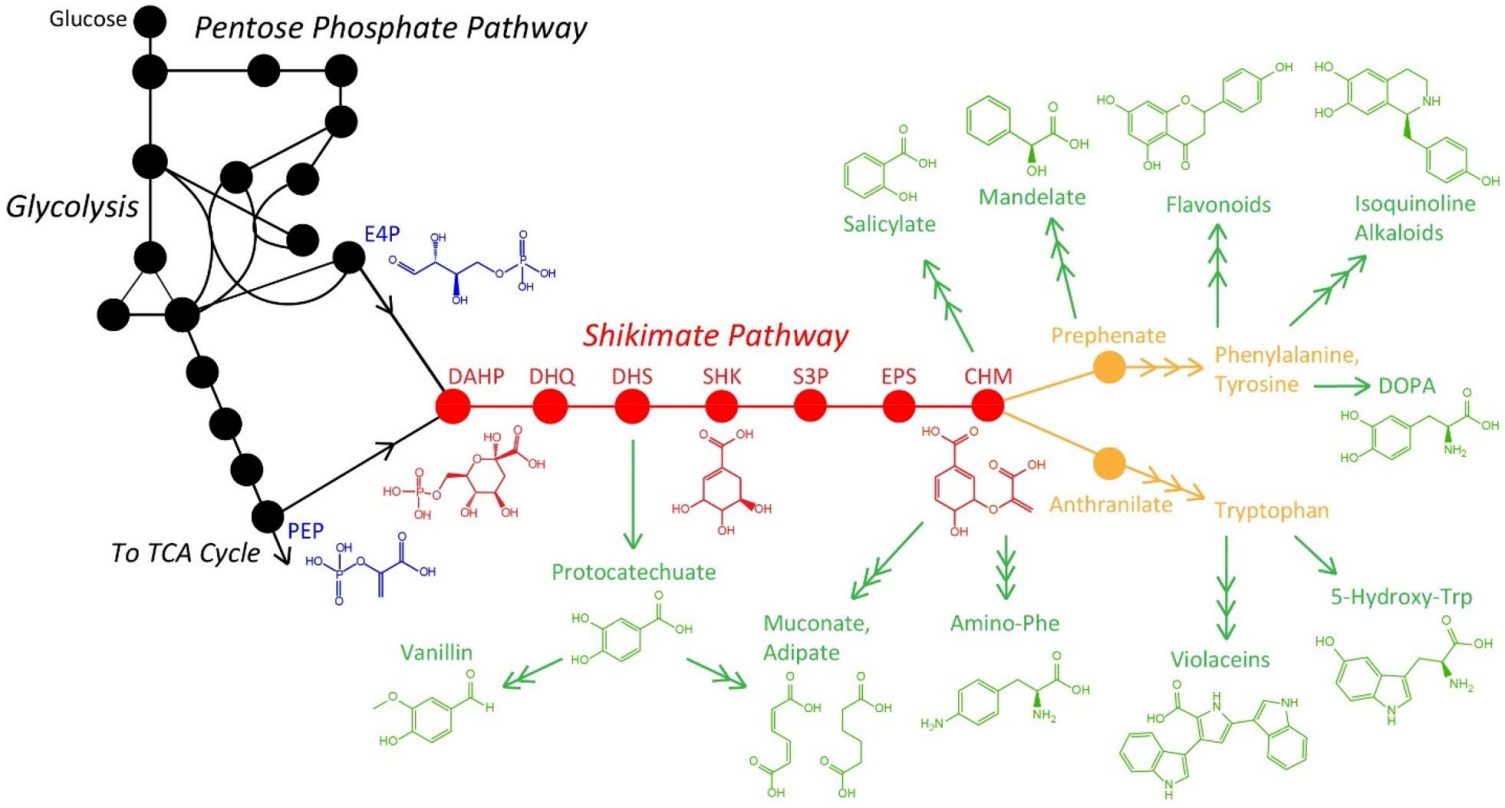
The aromatic amino acid biosynthesis pathway illustrated with several examples of engineered microbial products. Precursor molecules of erythrose-4-phosphate (E4P) and phosphoenolpyruvate (PEP) are shown in blue. Shikimate pathway metabolites are shown in red with abbreviations as follows: *DAHP* deoxy-d-arabinoheptulosonate 7-phosphate, *DHQ* 3-dehydroquinate, *DHS* 3-dehydroshikimate; *SHK* shikimate, *S3P* shikimate 3-phosphate, *EPS* 5-enolpyruvylshikimate-3-phosphate, *CHM* chorismate. Orange metabolites are native to non-auxotrophic microbes downstream of the chorismate branch point in aromatic amino acid biosynthesis. A representative sampling of metabolites that are non-native to most microbes but have been overproduced as a result of engineered heterologous expression are shown in green. Abbreviations used here are amino-Phe (4-amino-l-phenylalanine), DOPA (l-dihydroxyphenylalanine), and 5-hydroxy-Trp (5-hydroxy-l-tryptophan)

The core pathways for the biosynthesis of the three standard aromatic amino acids (AAAs)—tyrosine, phenylalanine, and tryptophan—are responsible for the production of the majority of natural aromatic metabolites (Huccetogullari et al. 2019). Although AAA biosynthesis is well-conserved within microorganisms and plants, it serves a broader range of purposes in plants and results in greater molecular diversity (Tzin and Galili 2010; Vogt 2010). AAA biosynthesis begins with the condensation of phosphoenolpyruvate (PEP) and erythrose 4-phosphate (E4P). This achieves the formation of 3-deoxy-d-arabinoheptulosonate 7-phosphate (DAHP), which is the first metabolite of the seven-step shikimate pathway for the production of chorismate (Herrmann and Weaver 1999) (Fig. 1). Chorismate is a precursor for all three standard AAAs and a key branch point for secondary aromatic metabolites. These secondary metabolites include large family classes such as flavonoids and isoquinoline alkaloids. Additionally, diverse commodity and high-value products have been produced by engineered microbial hosts, many of which will be described later in this review.

### Converting unwieldy pathways into modules

The last decade of metabolic engineering research has embraced the concept of modular pathway design and assembly. This has utility for the production of aromatic compounds given that their associated biosynthetic pathways can feature branch points, secreted intermediates, and inhibitory intermediates. Modularity is achieved in pathway design when a pathway is subdivided into distinct modules whose regulation and expression level can be independently tuned (Yadav et al. 2012). Modularity also allows for the interchangeability of pathway sections, which can enable product diversification. This can occur via substitution of downstream branches from a common platform pathway or via substitution of upstream pathways that generate different precursors for promiscuous enzymes that are located within downstream modules. In the production of aromatic compounds, modularity is more often used to optimize titers of key molecules that are entry points within a product class such that different downstream modules can be harnessed to achieve overproduction of a specific member of that class. Examples of aromatic biosynthesis pathway modules are described in the “Compartmentalization and co-culturing” sections that follow.

### Placing pathway modules within specialized compartments

**Fig. 2:**
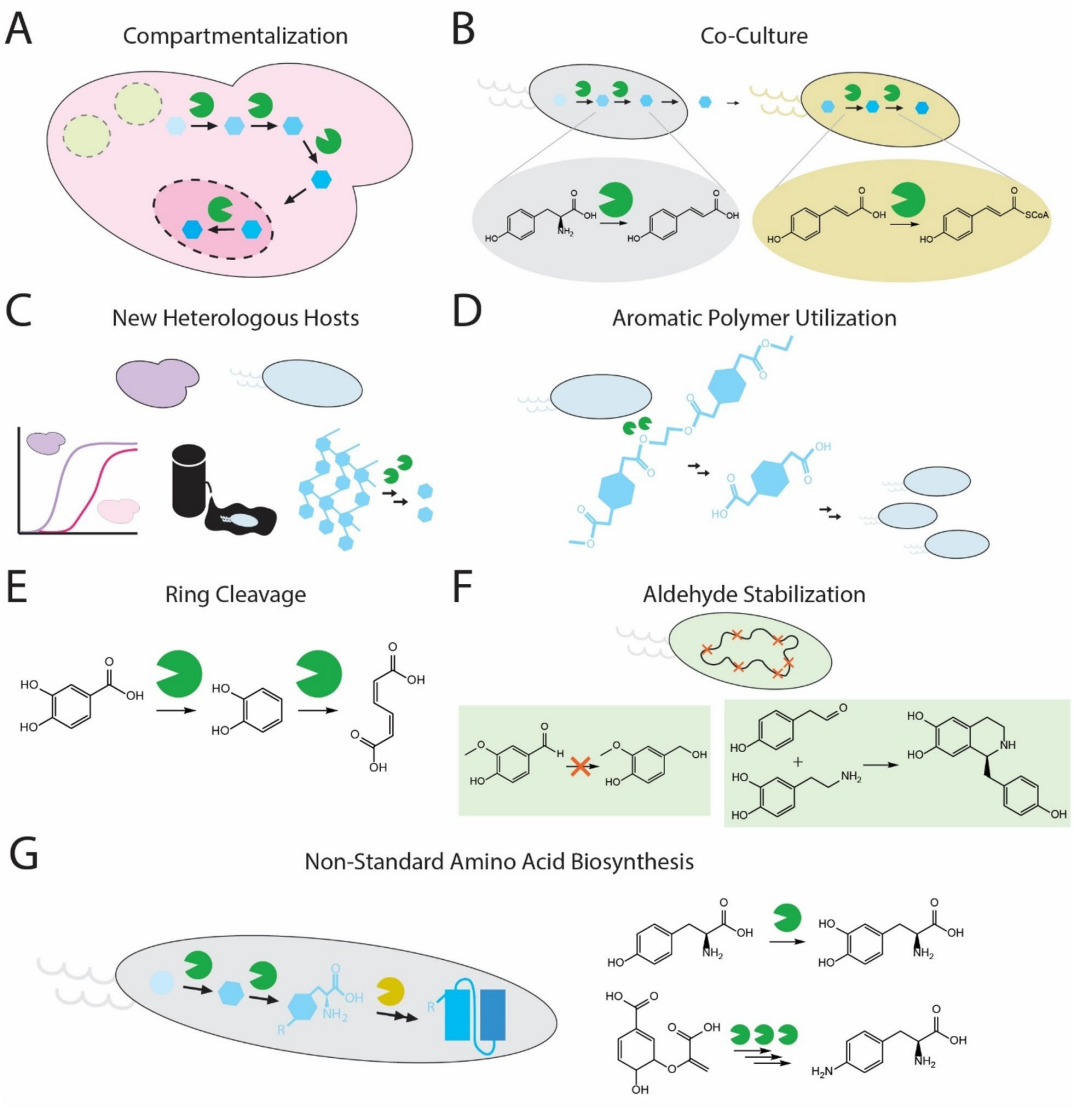
Emerging strategies used to improve the production of aromatic compounds in engineered microbes. **A** Internal cellular compartments can be used in eukaryotic microbes to target specific pathway modules. **B** Cultivation of two different organisms in one pot, also known as co-culturing, can be used to achieve division of labor between organisms that express distinct pathway modules. **C** New heterologous hosts are emerging for aromatic compound production given natural tendencies they may have for specific pathways and products. **D** A broader range of aromatic polymers are being broken down and utilized or valorized by microbes. **E** Aromatic compounds can be produced as intermediates with designed ring cleavage to achieve monomer units for important polymers. **F** Aldehyde stabilization has improved production of many different aldehyde or aldehyde-derived products across different organisms and research groups. **G** Aromatic non-standard amino acids are attractive emerging targets for biosynthesis given the ultra-high-value applications of the proteins that they can be incorporated within

As the complexity of synthetic pathways continues to grow, optimizing production titers can be limited by potential cross-talk of exogenous enzymes and native cellular responses (Lee et al. 2011). The co-localization of enzymes by covalent or non-covalent tethering in the cytosol is a common strategy to help potentially eliminate the escape of pathway intermediates which can reduce undesired side products. For eukaryotes, subcellular compartments allow for the physical separation of metabolic pathways within membrane organelles which can help ensure limitation of side reactions, toxicity, redox state and secretion of pathway intermediates (Sampson and Bobik 2008; Lee et al. 2011; Ayer et al. 2013; Hammer and Avalos 2017). This natural metabolic compartmentalization approach can be applied to aromatic biosynthesis pathways to increase flux to desired branches or isolate harmful reactions (Fig. 2A). One such compartment is the peroxisome, as the peroxisomal membrane has size-dependent permeability that allows free access for small hydrophilic molecules but presents a barrier for bulky co-factors (NAD/H, NADP/H, CoA) (Antonenkov et al. 2004). An enhanced, noncleaved, and tripeptide C-terminal peroxisomal targeting signal type 1 (ePTS1) in *Saccharomyces cerevisiae* was identified for rapid sequestering of non-native cargo proteins. The ePTS1 was applied to signal a two-enzyme pathway module into peroxisomes for the production of the green pigment prodeoxyviolacein (PDV) from tryptophan. The resulting recruitment of tagged enzymes into the peroxisome saw a 35% increase in PDV production compared to the nontagged controls and a decrease in the spontaneous by-product, chromopyrrolic acid (DeLoache et al. 2016). A similar strategy was used to increase mandelic acid titers from chorismate in *S*. *cerevisiae* through the compartmentalization of three enzymes in the mitochondria or peroxisomes (Reifenrath et al. 2018). Mandelic acid is used in the cosmetic industry and in the production of pharmaceutically active compounds. Enzymes targeted into the peroxisomes utilized the same ePTS1 targeting sequence as previously mentioned. Compartmentalization of enzymes into the mitochondria was achieved by using a N-terminal mitochondrial targeting sequence (MTS), consisting of less than 55 amino acids (Westermann and Neupert 2000). Both works suggest compartmentalization as a possible tool for increasing local concentrations of substrate and decreasing off-pathway by-product formation. Work has also been conducted to demonstrate that compartmentalization can mitigate toxicity by sequestering toxic proteins. High norcoclaurine synthase activity is required for efficient production of norcoclaurine, a key branch point intermediate in the biosynthesis of many benzylisoquinoline alkaloids (Nakagawa et al. 2011). However, the most active variant of norcoclaurine synthase is toxic when expressed in the cytosol of *S. cerevisiae*. Norcoclaurine titers from tyrosine were increased in *S*. *cerevisiae* through the use of the ePTS1 tag that targeted the toxic norcoclaurine synthase into the peroxisome to alleviate cytotoxicity (Grewal et al. 2021).

The concept of compartmentalization has also been expanded upon outside of native cellular compartments. Artificial vesicles derived from the endoplasmic reticulum (ER) have been used to form metabolic organelles for compartmentalization. This was achieved via the fusion of metabolic enzymes to Zera, a synthetic peptide composed of an N-terminal signal peptide, for the production of *cis,cis*-muconic acid (Reifenrath et al. 2020). Although the results showed lower enzymatic activity and muconic acid titers as compared to the cytosolic pathway, the utilization of ER-derived metabolic vesicles offers novel possibilities for metabolic engineering in yeast cells. Light-based inducible synthetic organelles have also been utilized in *S. cerevisiae* on the deoxyviolacein pathway as a model metabolic pathway. The light-switchable active enzyme clustering was shown to enhance product formation 6-fold and product specificity 18-fold (Zhao et al. 2019) The concept of harnessing selectively permeable barriers within cells to limit endogenous cross-talk can be extended further by applying it across two different strains.

### Splitting pathway modules across different strains

Metabolic burden, imposed by the expression of multiple enzymes or by feedback inhibition, can decrease the efficiency of aromatic compound production in microbial hosts. If the desired intermediates can be exchanged freely between microbes, division of labor through co-culturing is a potential option to circumvent these issues (Fig. 2B). This technique enables individual microbes in a consortium to express fewer enzymes by splitting a pathway across multiple strains. Besides decreasing burden, co-culturing is in many ways the bacterial analogy to the compartmentalization strategy demonstrated in eukaryotic microbes given that bacteria do not generally contain compartments (with the exception of a limited number of bacterial microcompartments that are not known to permit transport of aromatic compounds). The production of aromatic compounds through co-culturing remains relatively underexplored, with the first *Escherichia coli* co-culture experiment for flavonoid production published only 5 years ago (Jones et al. 2016). The optimized co-culture produced 40.7 mg/L of flavan-3-ols; a 970-fold improvement over previous monoculture productions. The increased titer in this study demonstrated a potential advantage for product formation through division of labor. The choice to co-culture strains expands the breadth of engineering design considerations at the cellular and process levels. For example, in the previously mentioned production of flavan-3-ols, parameters such as inoculation ratio, induction temperature, and strain compatibility had to be considered to achieve maximal titers. Despite these additional considerations, progress has been made on the production of aromatic products using *E. coli* co-cultures, and higher titers than were previously achieved through monoculture are now possible for certain products. Co-culturing has also led to product formation when the product was undetectable in an engineered monoculture. This was the case for the biosynthesis of specific pyranoanthocyanins that contribute to the taste and color of wine (Akdemir et al. 2019). Improved titers were achieved, however, optimized conditions only resulted in a final concentration of 19.5 mg/L after 7 days.

While division of labor through co-culturing can be beneficial, there are limitations that must be considered. The inefficiencies of transporting intermediates between microbes could significantly restrict the productivity of a co-culture. Not only is it difficult to control efflux systems (Zhou et al. 2012), but their expression can also burden the cell (Turner and Dunlop 2014). Also, some important lipophilic or activated pathway intermediates and co-factors, such as specific CoA species and phosphorylated molecules, have limited transport capabilities across the cell membrane (Jones et al. 2017; Jawed et al. 2019). Culture resources will also be divided when a pathway is split between two strains. Without adequate concentrations of necessary nutrients and careful optimization, the co-culture will not be stable. A theoretical model has been generated to predict when the aforementioned limitations posed by co-culture will be less burdensome than expressing a pathway in monoculture (Tsoi et al. 2018).

Besides division of labor, another benefit of co-culturing is that metabolites can be physically separated from enzymes they inhibit. For example, the tyrosine ammonia lyase (TAL) enzyme may be inhibited by coumaroyl-CoA (Santos et al. 2011). Researchers have overcome this issue by separating the pathway across two strains at the *p-*coumaric acid node, resulting in an upstream producer and a downstream utilizer. An example of this technique was demonstrated through the co-culture production of the curcuminoid bisdemethoxycurcumin, which has antioxidant and antibiotic properties. Its production in co-culture with glucose as the sole carbon source resulted in approximately 60% higher titers than monoculture simply by separating the production pathway between two strains at the *p-*coumaric acid node (Fang et al. 2018). In another study, a similar split in the pathway was conducted through co-culture to produce the therapeutic compound sakuranetin that reported the highest concentration produced compared to other *E. coli* production methods at 79 mg/L after bioreactor scale-up (Wang et al. 2020). By physically separating the TAL enzyme and coumaroyl-CoA, TAL inhibition by metabolites will no longer be a major barrier to achieving the maximum potential titer of the desired product.

Other co-culturing strategies have emerged recently that have been applied to aromatic compound biosynthesis. Researchers have created additional controls for the ratio of two microbes by employing quorum sensing. By splitting the path for naringenin production after *p-*coumaric acid production, a balance must be struck between its production and utilization to maximize titer. Therefore, a quorum-sensing circuit was created to use *N*-acyl homoserine lactone production to down regulate the growth of the *p*-coumaric acid producing strain and trigger malonyl-CoA accumulation in the *p*-coumaric acid utilizing strain (Dinh et al. 2020). Also, the use of *E. coli* polycultures have proven to produce aromatic compounds from complicated processes with increased product titers. The complex process of expressing 15 exogenous enzymes to produce the red pigment anthocyanidin-3-*O*-glucoside callistephin from glucose was successfully implemented for the first time outside of a plant using an *E. coli* polyculture, even though the final titer was only 9.5 mg/L (Jones et al. 2017). A polyculture again proved useful for the non-linear production pathway of rosmarinic acid, a polyphenol with both pharmaceutical and nutraceutical properties. Through this technique, researchers achieved a 38-fold titer increase compared to monoculture (Li et al. 2019). There is already significant promise for increased production capabilities by splitting pathways between *E. coli* strains. However, it has also been demonstrated that it is not only possible, but beneficial in some cases to move from co-cultures using exclusively *E. coli* strains to co-cultures between *E. coli* and *S. cerevisiae* or even purely *S. cerevisiae* co-cultures (Du et al. 2020; Yuan et al. 2020). As more knowledge is gained about other microbes, production could be further improved by splitting a pathway between multiple species that are better suited for specific steps. Similarly, a full pathway could be transferred to a less conventional host to improve aromatic compound production while still using monoculture, which will be further discussed below.

### Introducing heterologous pathways into new hosts

Many milestones in AAA biosynthetic pathway development were achieved in model *E. coli* and *S. cerevisiae* strains due to genetic tractability, genome annotation, and tools for gene expression (Ji et al. 2018). However, there may be other organisms better suited for overproduction of specific metabolites due to factors such as (i) elevated carbon flux through certain pathways; (ii) the ability to utilize non-conventional feedstocks; (iii) improved expression of plant-derived enzymes; (iv) improved growth rates under desired reactor conditions, and (v) increased tolerance of aromatic products (Fig. 2C). Here we describe a few other organisms that have recently been engineered to produce derivatives of AAA biosynthesis.

A yeast alternative to *S. cerevisiae* that has shown promising qualities for metabolite overproduction is *Kluyveromyces marxianus*. As a crab-tree negative yeast, diversion of flux from glucose to ethanol is not a concern, making it a promising choice for biomass accumulation. Its high production capabilities are coupled with a high secretory capacity, giving the strain potential for harvesting desired products or working in co-culture (Lin et al. 2017). Thus far, *K. marxianus* has been used as a host for production of aromatic compounds such as 2-phenylethanol (Kim et al. 2014) and, more recently, the non-aromatic carotenoid product astaxanthin (Lin et al. 2017). *K. marxianus* was also used in conjunction with the anaerobic fungus *Piromyces indianae* in a novel two-stage bioprocess to produce 2-phenylethanol from lignocellulosic biomass that was hydrolyzed by *P. indianae* (Hillman et al. 2021). As progress continues to be made in synthetic biology and the capabilities of genetic manipulation in *K. marxianus* are improved, there is potential for it to become a much more common strain for biosynthesis of AAA derivatives.

Another yeast strain that has emerged as a promising host for biosynthesis of aromatic compounds is the oleaginous yeast *Yarrowia lipolytica*. Like *K. marxianus*, *Y. lipolytica* has also been engineered to produce 2-phenylethanol, achieving titers of 2 g/L that were competitive with other yeasts as of 2013 (Celińska et al. 2013). In the past year, *Y. lipolytica* has been engineered to produce *p*-coumaroyl-CoA derived products such as resveratrol and naringenin (Palmer et al. 2020; Sáez-Sáez et al. 2020), as well as tryptophan-derived dyes such as violacein and deoxyviolacein (Gu et al. 2020) or protodeoxyviolaceinic acid (Larroude et al. 2020). As an oleaginous yeast, *Y. lipolytica* has high flux through lipid biosynthesis. Additionally, industrial know how around this organism has increased during the last decade. However, its relevance to aromatic compounds may be highest for its catabolism of aromatic compounds to produce fatty acid derivatives. Recent work has screened numerous oleaginous yeasts for these abilities (Yaguchi et al. 2020).

A cyanobacterium, *Synechocystis* sp. PCC 6803, has been shown to successfully express *p*-coumarate-3-hydroxylase, which is a cytochrome P450 enzyme that has traditionally been difficult to express in other organisms (Xue et al. 2014). This allowed production of caffeic acid from *p-*coumaric acid. Caffeic acid comes from plants and has antioxidant and anticancer abilities among other therapeutic properties. The study suggests it could be easier for photosynthetic *Synechocystis* to express proteins similar to cytochrome P450 enzymes because many already exist in cyanobacteria. Additionally, their photosynthetic capabilities may make them more amenable to expression of plant-derived enzymes (Xue et al. 2014). The ability of *Synechocystis* to produce and excrete precursors to phenylpropanoids, like *p*-coumaric acid, cinnamic acid, and caffeic acid, has already been demonstrated (Brey et al. 2020).

*Corynebacterium glutamicum* is well-known as an amino acid overproducer with mature genome-scale metabolic models and past flux balance analysis (Wittmann 2010). When using soil bacteria to produce aromatic compounds, one may have to inactivate complex catabolic networks that are capable of breaking down aromatic compounds (Kallscheuer et al. 2016). Yet in some cases, the catabolism of aromatic compounds is a phenotype that is strongly sought after because it increases the likelihood of utilizing aromatic feedstocks. In the case of *C. glutamicum,* carbon flux is elevated from central metabolism to various amino acid biosynthesis, and thus it may be advantageous to design pathways from simple carbon sources while knocking out aromatic catabolic networks. It was shown that by knocking out 4 gene clusters comprising 21 enzymes in the aromatic catabolic network, several stilbenes and (2*S*)-flavanones were able to be produced by this strain. Similarly, in a continuation of this work, additional catabolic pathways were knocked out to form several hydroxybenzoates, which following oxidation form industrially relevant hydroxybenzoic acids (Kallscheuer and Marienhagen 2018).

While our list of organisms used for production of microbial compounds is not intended to be comprehensive, we will mention one other important bacterium, *Pseudomonas putida*. Considerable work was reported in the early 2000s to engineer this microbe to produce aromatic compounds. These include the biosynthesis of phenol via the action of a heterologous tyrosine phenol lyase (Wierckx et al. 2005), cinnamic acid from glucose or glycerol (Nijkamp et al. 2005), *p*-coumaric acid (Nijkamp et al. 2007), *p*-hydroxybenzoic acid (Verhoef et al. 2007; Meijnen et al. 2011), *p*-hydroxystyrene (Verhoef et al. 2009), and anthranilate (Kuepper et al. 2015). It has been used for the conversion of lignin breakdown products to the aliphatic polymer precursor adipic acid (Niu et al. 2020) and to polyhydroxyalkanoate biopolymers (Salvachúa et al. 2020). Additionally, *P. putida* was recently engineered for the production of the aromatic pigment, indigoidine, though this pathway extends from l-glutamine (Eng et al. 2021). Overall, the choice of an optimal and less-conventional heterologous host largely depends on the target aromatic compound and the desired process conditions, but evidence suggests that alternative hosts could aid in the titer optimization required for industrial feasibility (Table 1).

**Table 1:** Relevant attributes and notable products of unconventional hosts for AAA biosynthesis

Species	Attributes	Notable products
*K. marxianus*	Withstands temperatures up to 52 °C Utilizes xylose and lactose as carbon sources Fast growth rate	2-Phenylethanol Astaxanthin
*Y. lipolytica*	Catabolizes aromatic compounds High flux through lipid biosynthesis	2-Phenylethanol Resveratrol Naringenin Violacein Deoxyviolacein Protodeoxyviolaceinic acid
*Synechocystis*	Photosynthetic Large NADH pool Natural secretion of aromatic products	Caffeic acid *p*-Coumaric acid Cinnamic acid
*C. glutamicum*	Amino acid overproducer Catabolizes aromatic compounds	Stilbenes (2*S*)-Flavanones Hydroxybenzoates
*P. putida*	Solvent tolerant Catabolizes aromatic compounds Highly reductive	Phenol Cinnamic acid *p*-Coumaric acid *p*-Hydroxybenzoic acid *p*-Hydroxystyrene Anthranilate Adipic acid Polyhydroxyalkanoate biopolymers Indigoidine

### Harnessing natural and synthetic aromatic polymers as feedstocks

Whether aromatic polymers are of natural or synthetic origin, they are often discarded in the environment or incinerated due to their recalcitrance for (re)use. Alternatively, their aromatic monomers could be utilized as atom-economical feedstocks for engineered microbes, particularly if the goal is to form aromatic bioproducts (Fig. 2D). One notable example is lignin, which is the most abundant aromatic polymer that occurs in nature. Lignin is a non-edible and multi-unit aromatic heteropolymer that provides plants their tough exterior and structure. As of 1998, 99% of lignin obtained during paper production was burned for energy (Thielemans et al. 2002). With the rise in demand for biofuels, biorefineries that convert cellulosic biomass into fuels will generate a greater abundance of lignin than needed to power the operation (Ragauskas et al. 2014). Considerable work has been performed on degradation pathways for lignin-based aromatics; however, the complex structure, variety of linkages, and recalcitrance of lignin still presents a challenging task for depolymerization efforts. Lignin can be classified as native or technical lignin. Native lignin refers to the original lignin structure in lignocellulose and technical lignin refers to modified lignin which has been extracted from biomass or recovered from industrial processes. Various fractionation methods have been developed for the isolation of technical lignin from lignocellulose (Chakar and Ragauskas 2004; Pinkert et al. 2009; Bunzel et al. 2011; Kim et al. 2016; Sun et al. 2018b). Technical lignin (Kraft, soda, sulfite, organosolv, ionic liquid, deep eutectic solvent-extracted lignin) offer aromatic hydroxyl groups as a major constituents (Chio et al. 2019). A considerable amount of research has focused on conventional catalytic depolymerization approaches for the potential valorization of lignins, with particular focus for its valorization into value-added phenolic monomers (Zakzeski et al. 2010; Zhang and Wang 2020; Liu et al. 2020b). Additionally, the emerging strategy of reductive catalytic fractionation of lignin presents a promising method for phenolic monomer production (Ma et al. 2015; Van Den Bosch et al. 2015; Liu et al. 2020b). Bio-catalytic processes for lignin depolymerization and valorization have also been studied to enable lignin processing under milder conditions (Lancefield et al. 2016; Picart et al. 2017; Xu et al. 2018; Bugg et al. 2020). However, efficient breakdown of lignin remains a challenge.

When lignin is broken down biologically, major degradation products such as ferulic acid, *p-*coumaric acid, and syringic acid, offer a great deal of synthetic pathway versatility. However, the complexity of lignin product mixtures presents a potential problem as isolating pure chemicals from a diverse input stream is challenging. One strategy to alleviate this hurdle is the concept of metabolic funneling, which converts a broad mixture into a smaller pool of central platform intermediates that can then be transformed into desired products (Linger et al. 2014; Schutyser et al. 2018). Alternatively, the relatively high cost and inefficiencies of biological or synthetic lignin degradation strategies could be more commercially palatable if coupled to production of higher value materials or bioactive products. The high-value applications and growing markets of aromatic secondary metabolites, as well as the technical advances of the last decade, create several possibilities for harnessing aromatic feedstock for such “upcycling”.

Similar principles can apply to synthetic aromatic polymers that have accumulated in the environment, such as the common consumer plastic polyethylene terephthalate (PET) (Koshti et al. 2018). As the consumer demand for PET continues to increase, so do the environmental concerns surrounding plastic waste. The majority of high market-share plastics are obtained from the use of nonrenewable and ecologically damaging petroleum/natural gas feedstocks and processing techniques. The high environmental persistence, low recycling rates, and the loss of mechanical properties of plastic when recycled additionally intensify these concerns (Jankauskaite et al. 2008; Hong 2017; EPA 2018; Eriksen et al. 2019). Bio-based alternatives to PET have gained traction to address the sustainability of raw materials. Polyethylene furanoate (PEF) contains similar polyester linkages and is produced from 100% renewable materials. However, PEF is also not biodegradable and thus requires methods of mechanical or chemical recycling to avoid waste. The depolymerization of PET is well-established catalytically, with the glycolysis of PET yielding a bis(2-hydroxyethyl) terephthalate monomer (Raheem et al. 2019; Xin et al. 2021). Although extensive work has been done to find greener catalytic routes for PET depolymerization, problems with long reaction times, low yields, and undesirable conditions still remain (Al-Sabagh et al. 2016). Alternatively, biocatalytic routes for the depolymerization of PET or PEF to their respective monomer substrates of terephthalic acid (TPA) or furandicarboxylate (FDCA) can be achieved. In the field of enzymatic plastic degradation, there are two main families of enzymes; single plastic-specific hydrolases and the more promiscuous cutinases. Since the first discovery of PET hydrolases in *Thermobifida fusca* in 2005, great strides have been made for more efficient depolymerization methods (Müller et al. 2005). Hydrolytic enzymes, PETase (PET hydrolase) and MHETase (Mono(2-hydroxyethyl) terephthalic acid (MHET) hydrolase), from a novel bacterium, *Ideonella sakaiensis*, were discovered that are capable of using PET as its major energy and carbon source (Yoshida et al. 2016). This PET degradation pathway of PETase and MHETase for the production of TPA has been extensively studied over the past 5 years with activity also shown on PEF as a substrate for the production of FDCA (Austin et al. 2018; Knott et al. 2020). PET hydrolases are versatile polyesterases that have the advantage of high thermostability in terms of reaction speed and durability (Kawai et al. 2020).

As biological depolymerization of PET into monomer units of TPA has become increasingly viable, researchers have reported bioconversions that harness PET or TPA directly. Gallic acid, pyrogallol, catechol, muconic acid, and vanillic acid have been synthesized from TPA using a whole-cell catalysis comprising engineered *E. coli* expressing necessary metabolic enzymes (Kim et al. 2019). Additionally, a one-pot chemo-bioprocess involving chemical glycolysis, enzymatic hydrolysis, and whole-cell bioconversion has been shown for the production of protocatechuic acid (Kim et al. 2021). The chemical glycolysis of PET utilized a biocompatible betaine catalyst for the production of bis(2-hydroxyethyl) terephthalate that can be converted to TPA via mutant PETases. As in the case of lignin upgrading, many researchers seek to identify microbes and associated pathways that can utilize these aromatic polymers for growth. Entry of carbon flux into central metabolic pathways alleviates the need for another carbon source and enables redirection of flux towards theoretically any bioproduct, including high-volume and low-cost applications such as fuels, commodity chemicals, and animal food additives. However, these strategies commonly feature the destruction of the aromatic ring. We believe a useful alternative worth exploring is the preservation of aromatic structures by channeling breakdown products to high-value secondary aromatic metabolites using bioconversion processes. Overall, the prospect of lignin and plastic upcycling offers strategies for utilization of sustainable feedstocks in novel synthetic pathways.

### Cleaving aromatic rings to form valuable compounds

In some cases, the cleavage of an aromatic precursor is well-justified, particularly during the biosynthesis of monomers that are currently used at high volumes to produce polymers (Fig. 2E). Although the final products are no longer aromatic, the dependence of these routes on genes involved in aromatic metabolism should interest readers given increased opportunities to expand the microbial product portfolio. In addition to the use of aromatic catabolic pathways mentioned earlier, innovative de novo biosynthetic pathway designs have been reported recently that couple AAA biosynthesis pathways to a ring cleavage reaction pathway. For example, *cis,cis*-muconic acid is an important precursor for the production of adipic acid, TPA, 1,6-hexanediol, and pharmaceuticals. Its production in *E. coli* was proposed via a non-native pathway utilizing the ring opening of catechol from a chorismate intermediate (Sengupta et al. 2015). This work was expanded on with the implementation of five non-natural pathways, starting from chorismate, tyrosine, and 3-dehydroshikimate to generate a synthetic “metabolic funnel” to the catechol aromatic ring opening (Thompson et al. 2017). Additional advances in muconic acid production in subsequent years have been made through AAA gene knockouts as well as the overexpression of a fusion protein to increase chorismate flux to isochorismate (Fujiwara et al. 2018). Similar to the pathway for muconic acid production, a synthetic pathway was constructed in *E. coli* for the synthesis of maleate, a high-valued building block of polymer materials and pharmaceuticals. The benzene ring cleavage reaction from aromatic compound-degrading bacteria is then used to produce maleate. This pathway design with further optimization under fed-batch culture conditions enabled the production of 4.5 g/L of maleate, a 268-fold improvement compared with the titer generated by the wild-type *E. coli* strain carrying the entire maleate biosynthetic pathway (Noda et al. 2017). An alternative maleate biosynthesis pathway from glycerol and using salicylate as an intermediate has also been shown (Sheng et al. 2021). This ring-opening strategy has been implemented to catabolize protocatechuate, a common intermediate in lignin degradation, to provide a platform for the conversion of lignin-derived aromatics. This heterologous pathway using protocatechuate as a model compound allowed for cell growth with protocatechuate as the sole source of carbon and energy (Clarkson et al. 2017). A biosynthesis pathway for the production of glutaconic acid, a potential precursor for the production of nylons and biodegradable polymers, has also been introduced into *E. coli* again using the heterologous catechol ring opening pathway (Sun et al. 2018a). Collectively, these works illustrate that aromatic ring cleavage presents an alternative fate for biosynthesized aromatic compounds, which expands their relevance to industrially significant non-aromatic compounds.

### Controlling the fate of aromatic aldehydes in live cells

In the pursuit of engineering de novo product biosynthesis or whole-cell bioconversions, aldehydes had been overlooked for a long time despite large and growing markets for these products in high-value applications such as flavors and fragrances, where there is also consumer preference for biologically derived products (Kunjapur and Prather 2015; Sulzbach and Kunjapur 2020; Zhou et al. 2020; Kazimírová and Rebroš 2021). These include products such as vanillin (Walton et al. 2003), benzaldehyde (Opgrande et al. 2000), and piperonal (also known as heliotropin) (Gatfield 1999). Owing to their unique reactivity, aldehydes can also be useful as intermediates including to non-natural compounds such as the chiral pharmaceutical intermediate (*R*)-phenylacetylcarbinol (R-PAC) (Tripathi et al. 1997). Limited focus on aromatic aldehyde products was, in part, due to few known promiscuous biocatalysts for their synthesis, short stability of aldehydes in the presence of metabolically active microbial cells, and inherent toxicity of aldehydes to microbial cells. However, as described in the following paragraphs, several of these challenges have recently been overcome, allowing for aldehyde biosynthesis and retention as well as an expansion of aldehyde-based enzymatic transformations in live cells (Fig. 2F).

Many aromatic metabolites that are downstream of shikimate contain one or more carboxylic acid functional groups as a ring substituent or on a sidechain. An ideal enzyme for aldehyde biogenesis would catalyze chemoselective and partial reduction of this group with broad substrate tolerance while maintaining some orthogonality to primary metabolic pathways to avoid toxicity. Carboxylic acid reductases (CARs) are an enzyme class where the first notable member was characterized in recombinant hosts in the early 2000s (Butler and Kunjapur 2020). At that time, the CAR from *Nocardia iowensis* (NiCAR) was shown to exhibit broad substrate specificity for aromatic carboxylic acids (He et al. 2004; Venkitasubramanian et al. 2007). Because heterologous expression of CARs in *E. coli* enabled facile bioconversion of externally supplied carboxylic acids to their corresponding alcohols, one remaining challenge was to mitigate or prevent the overreduction that was believed to be due to many native redundant aldehyde reductases. Deletion of the genes that encode a limited number of candidate aldehyde reductases across two enzyme superfamilies, aldo–keto reductases (*dkgA, dkgB, yeaE*) and alcohol dehydrogenases (*yqhD, yahK, yjgB*), has proven very effective at stabilizing a wide range of aromatic aldehydes in the presence of CAR overexpression. The resulting *E. coli* strain, named “RARE” for its attribute of “Reduced aromatic aldehyde reduction”, was initially demonstrated to achieve efficient bioconversion of benzoic acid to benzaldehyde, de novo biosynthesis of vanillin, and bioconversion of benzaldehyde to (R)-PAC (Kunjapur et al. 2014). This de novo vanillin biosynthesis pathway in *E. coli* has since been further optimized at the step of *O*-methylation through deregulation of *S*-adenosylmethionine biosynthesis (Kunjapur et al. 2016) and construction of a genetically encoded vanillate biosensor to screen *O*-methyltransferases for increased activity on the precursor protocatechuate (Kunjapur and Prather 2019). Additional biosynthetic pathways or bioconversion processes that have been enabled by the retention of aromatic aldehydes in live microbial cells are listed in Table 2.

**Table 2:** Novel biosynthetic pathways or bioconversions based on stabilization of aromatic aldehydes

Feedstock	Product	References
Glucose	Vanillin	Kunjapur et al. (2014)
Benzaldehyde and glucose	(*R*)-Phenylacetylcarbinol	Kunjapur et al. (2014)
Piperonylic acid	Piperonal	Schwendenwein et al. (2016)
Phenylalanine	Benzaldehyde Benzyl amine	Liu et al. (2020a), Zhu et al. (2020)
Glucose	Norcoclaurine (*S*)-reticuline	Pyne et al. (2020)
Isoeugenol	Vanillic acid Muconic acid	Chen et al. (2021)
Coumaric acid or ferulic acid	Hydroxyphenylacetic acid Homovanillic acid	Zhao et al. (2021)
Polyethylene terephthalate	Vanillin	Sadler and Wallace (2021)

The value of aromatic aldehyde stabilization has also been shown recently in yeast. The tetrahydroisoquinoline structural moiety forms the basis of over 3000 natural products, of which the benzylisoquinoline alkaloids mentioned earlier are a subset. Key to the synthesis of tetrahydroisoquinolines is the Pictet–Spengler condensation between an aryl amine and a carbonyl compound (Pyne et al. 2020). Much of the structural diversity of this category of alkaloid products arises from four aldehyde species: 4-hydroxyphenylacetaldehyde, 4-hydroxydihydrocinnamaldehyde, protocatechuic aldehyde, and secologanin. The condensation of 4-hydroxyphenylacetaldehyde and dopamine produces the entry molecule norcoclaurine, which can be converted to the downstream scaffold molecule (*S*)-reticuline. To overcome undesired oxidation/reduction of 4-hydroxyphenylacetaldehyde, a functionally redundant subset of seven oxidoreductases (Ari1, Adh6, Ypr1, Ydr541c, Aad3, Gre2, and Hfd1) were deleted from the genome of *S. cerevisiae*. The resulting stabilization of 4-hydroxyphenylacetaldehyde was combined with improvements to dopamine biosynthesis to achieve titers of norcoclaurine exceeding 1 g/L.

Recent efforts have strived to improve aromatic aldehyde stabilization by avoiding the need for gene knockouts and by circumventing aromatic aldehyde toxicity. One strategy attempted to lower by-product alcohol formation resulting from *E. coli* host cells by increasing reaction temperature to 50 °C (Ni et al. 2018). The proposed concept is to then identify desired biocatalysts for aldehyde synthesis from thermophilic hosts for heterologous expression in *E. coli* during elevated reaction temperatures. A complementary approach was implemented in yeast to minimize redox byproducts of supplemented benzaldehyde during R-PAC bioconversion (Bruder and Boles 2017). Here, the CAR from *N. iowensis* was expressed in yeast to eliminate accumulation of benzoic acid. Furthermore, based on the hypothesis that NADPH-dependent aldehyde reductases play a more dominant role in benzaldehyde reduction under the conditions tested, the native glucose-6-phosphate dehydrogenase enzyme (Zwf1) was deleted to lower cellular NADPH production. This method succeeded in lowering benzyl alcohol production and in increasing R-PAC titers. This approach could be useful for resting cell biocatalysis but is unlikely to work well during de novo biosynthesis using metabolically active cells.

To mitigate the issue of aromatic aldehyde toxicity, the aldehyde steady-state concentration has been lowered by fostering conditions for it to reversibly convert in an equilibrium between the carboxylic acid, aldehyde, and alcohol states (Bayer et al. 2017). An interesting observation noted in this study and in the original report describing the creation of the *E. coli* RARE strain is that oxidation of aldehydes to the carboxylate form is seen in the absence of CAR expression. Because CARs require ATP and NADPH co-factors to restore the aldehyde form, whereas endogenous aldehyde dehydrogenases are likely to use NAD^+^, simultaneous use of CAR and endogenous aldehyde oxidation creates a costly futile cycle. The unseen endogenous aldehyde oxidation occurring in the background of CAR expression is an unaddressed challenge for all cellular reactions that feature aldehyde accumulation after reduction catalyzed by CAR. Future research directions could seek to eliminate the endogenous oxidation of aromatic aldehydes that is observed in the absence of CAR expression by determining and deleting the associated aldehyde dehydrogenases.

### Biosynthesizing non-standard aromatic amino acids for protein engineering

An emerging frontier for the field of metabolic engineering is the biosynthesis of non-standard amino acids (nsAAs) (Völler and Budisa 2017), which are amino acids that are not among the 20 standard amino acids used by the ribosome for protein translation. The last few decades have featured demonstrations of incorporation of over 300 nsAAs within user-directed sites of target proteins using engineered aminoacyl-tRNA synthetases (de la Torre and Chin 2020). Notably, the majority of these nsAAs have been aromatic derivatives of phenylalanine and tyrosine (Dumas et al. 2015). nsAAs broaden the repertoire of biological chemistry available for targeted protein modulation in living systems for applications such as polypeptide conjugation (Chin et al. 2002; Wang et al. 2003; Deiters and Schultz 2005; Seitchik et al. 2012), polypeptide stimuli–response (Alfonta et al. 2003; Bose et al. 2006; Peters et al. 2009), artificial metalloenzyme engineering (Xie et al. 2007), and intrinsic biological containment of whole organisms (Mandell et al. 2015; Rovner et al. 2015; Kunjapur et al. 2018, 2021; Kohman et al. 2018; Parker and Kunjapur 2020).

Several distinct biocatalysts have been applied previously for nsAA synthesis, with some exhibiting substrate polyspecificity and most exhibiting reaction reversibility (Nagasawa et al. 1981; Taylor et al. 1998; Ward and Wohlgemuth 2010; Fesko 2016; Rowles et al. 2016). Biocatalysis usually achieves high enantiomeric excess, which is important for most applications that feature amino acids, including site-specific incorporation in live cells. Additionally, these chemoselective enzymes avoid the need for protection/deprotection strategies associated with traditional synthesis, which is important given the propensity of nsAAs to contain reactive functional groups on their sidechains (Almhjell et al. 2018). However, the need for enzyme purification, excess reactant concentrations to drive reactions forward, and product isolation has limited the use of biocatalysis.

The engineering of nsAA biosynthesis in live cells is an attractive option and may offer several important advantages over biocatalysis. One advantage is that live cells could integrate both steps of nsAA production and incorporation, resulting in one-pot fermentation and biomanufacturing process intensification. Another advantage of engineering nsAA biosynthesis is the ability to produce nsAAs on-demand and in diverse environmental contexts from available inexpensive precursors or simple carbon sources. Furthermore, nsAA biosynthesis can circumvent challenges of cellular uptake of nsAAs, increase intracellular pool sizes of nsAAs to improve aminoacylation, overcome thermodynamic barriers associated with nsAA-producing biocatalysts, and lower raw material cost. Several of these issues have been a hindrance to the use of nsAAs in industrial scale biomanufacturing but are beginning to be addressed (Fig. 2G).

The first report in which an nsAA was biosynthesized for the purpose of incorporation within proteins focused on a naturally occurring nsAA, *p*-amino-phenylalanine (amino-Phe) (Mehl et al. 2003), which was a well-known intermediate of the chloramphenicol biosynthesis pathway in *Streptomyces* species (Westlake and Vining 1969; Blanc et al. 1997). Transformation of *E. coli* with the *papABC* genetic operon resulted in conversion of the endogenous metabolite chorismate to *p*-aminophenylpyruvate. Endogenous aminotransferase reversibly led to the formation of amino-Phe, which was subsequently incorporated into a target protein based on suppression of an amber stop codon enabled by an engineered derivative of the tyrosyl-tRNA synthetase from *Methanocaldococcus jannaschii*. This pioneering effort was followed up on in recent years as labs identified applications for amino-Phe or sought to increase amino-Phe titers using metabolic engineering strategies. The utility of amino-Phe increased when a mild bioconjugation strategy was developed for aniline residues on proteins (Obermeyer et al. 2014). Strains that were capable of amino-Phe biosynthesis and utilization were then engineered to incorporate amino-Phe within an anti-HER2 single-chain variable fragment antibody for streamlined fluorophore labeling and imaging (Chen et al. 2018). In two other studies, amino-Phe biosynthesis was improved independently of incorporation technology given its diverse end uses. One study featured the use of *papABC* orthologs from *Pseudomonas fluorescens* and a feedback-resistant DAHP synthase, resulting in 4.4 g/L amino-Phe and a yield of 17% by mass during fed-batch culturing from glucose (Masuo et al. 2016). Another study investigated the effect of several changes, including glycerol utilization, overexpression of the *pabAB* operon from *C. glutamicum* in place of the *papA* gene from *S. venezuelae*, overexpression of the endogenous *aroFBL* genes, use of a host strain that is auxotrophic for tyrosine and phenylalanine and features additional copies of *tktA* and *glpX* genes, and deletion of the *tyrR* regulator (Mohammadi Nargesi et al. 2018). Collectively, these modifications enabled a titer of 5.47 g/L amino-Phe and a mass yield of 16% from glycerol.

To our knowledge, two other naturally occurring aromatic nsAAs have been targeted for de novo biosynthesis with the motivation of incorporation within proteins: l-dihydroxyphenylalanine (DOPA) and 5-hydroxytryptophan. As with amino-Phe, there are diverse end uses for DOPA that make it a desirable target for biosynthesis, including its role as an FDA-approved therapeutic for the treatment of Parkinson’s disease. Biosynthesis of DOPA requires just one enzymatic step downstream of tyrosine, which is catalyzed by the two-component flavin-dependent monooxygenase HpaBC. Genes encoding *hpaBC* are native to certain *E. coli* strains such as *E. coli* W and were initially implicated in the catabolism of hydroxyphenylacetic acid (Díaz et al. 2001). Metabolic engineering was first applied to DOPA biosynthesis in 2011, with the application of many standard interventions such as *tyrR* deletion, phosphotransferase system inactivation, feedback-resistant DAHP synthase overexpression, and overexpression of certain endogenous pathway genes (Muñoz et al. 2011). A later study applied the genome engineering technique of multiplex automated genome engineering (MAGE) to 23 genomic targets in *E. coli*, which increased titers to 8.67 g/L in fed-batch cultivation (Wei et al. 2016). More recently, titers as high as 25 g/L have been achieved by engineering the HpaB protein and using *E. coli* BL21 as a host (Fordjour et al. 2019). An alternative strategy of supplying catechol, pyruvate, and ammonia to *E. coli* whole cells that express tyrosine phenol lyase derived from *Erwinia herbicola* has led to comparable or superior DOPA titers (Zeng et al. 2019; Han et al. 2020). Both of these DOPA biosynthesis strategies have recently been used to achieve combined biosynthesis and incorporation in individual strains (Kim et al. 2018; Thyer et al. 2021). Lastly, biosynthesis and incorporation of 5-hydroxytryptophan, which occurs naturally in eukaryotes, was achieved using a promiscuous phenylalanine 4-hydroxylase from *Xanthomonas campestris*, an artificial tetrahydromonapterin co-factor recycling pathway, and an engineered tryptophanyl-tRNA synthetase from *S. cerevisiae* (Chen et al. 2020). This nsAA was used for bio-orthogonal conjugation of proteins using a rapid chemoselective azo-coupling reaction.

Overall, exciting strides have been taken in recent years to expand the set of AAAs that microbes can produce and then site-specifically incorporate within target proteins. One other notable effort related to nsAA biosynthesis is the recent elucidation of the biosynthesis of a terminal alkyne-containing amino acid (Marchand et al. 2019), but we do not discuss that further here given that it is not an aromatic compound. Given that efforts to date have demonstrated the biosynthesis of a limited number of naturally occurring nsAAs, an outstanding question in this emerging field is what breadth of useful natural or unnatural nsAAs could be attainable via metabolic engineering.

## Conclusions and future directions

While the production of aromatic compounds in microbes has long been of interest, innovations in roughly the last 5 years have driven the field in distinct directions. Conceptual advances in the subdivision and spatial arrangement of aromatic biosynthesis pathways have helped overcome inhibition and created new opportunities for engineering. Pathway engineering in diverse hosts has generated a plethora of options guided by industrial considerations such as rapid growth, substrate utilization, and tolerance of solvent or products. Much work underway is geared towards the utilization of polymeric aromatic feedstocks or their breakdown products. Innovative strategies have been designed to harness AAA biosynthesis pathways on route to non-aromatic products. Stabilization of aromatic aldehydes has enabled diverse biosynthesis and bioconversion processes that feature aldehydes as products or intermediates. Lastly, out-of-the-box applications are being pursued for the metabolic engineering of aromatic products, including engineering biosynthesis of aromatic nsAAs that can be site-specifically incorporated within proteins. Collectively, these developments suggest that research in aromatic compound biosynthesis is far from stagnating and promises to aid in the pursuit of an ever-growing set of objectives that benefit society.

In future research efforts, we expect to see innovative contexts in which biosynthesis pathways for aromatic compounds are shared among microbes in a consortium. We also expect to learn more about the molecular basis for aromatic product tolerance in unique microbes and to witness examples of partially transferring these traits to conventional hosts through heterologous expression. Improved enzymes for the degradation of recalcitrant polymer wastes are routinely being reported, and we expect greater integration with non-biological pre-treatment or catalytic strategies. Designed ring cleavage pathways for aromatic compounds hold high promise for forming monomers of new materials and we may see more of those materials synthesized and evaluated in the coming years. Additionally, future biosynthesis research efforts may access a greater diversity of aromatic nsAAs and may devise novel pathways that feature aromatic aldehydes as intermediates.

## Data Availability

Not applicable.
